# Impact of Early-Life Exposures to Infections, Antibiotics, and Vaccines on Perinatal and Long-term Health and Disease

**DOI:** 10.3389/fimmu.2017.00729

**Published:** 2017-06-23

**Authors:** Steven L. Raymond, Jaimar C. Rincon, James L. Wynn, Lyle L. Moldawer, Shawn D. Larson

**Affiliations:** ^1^Department of Surgery, University of Florida College of Medicine, Gainesville, FL, United States; ^2^Department of Pediatrics, University of Florida College of Medicine, Gainesville, FL, United States

**Keywords:** innate immunity, inflammation, infectious disease, sepsis, vaccination, immune agonists

## Abstract

Essentially, all neonates are exposed to infections, antibiotics, or vaccines early in their lives. This is especially true for those neonates born underweight or premature. In contrast to septic adults and children who are at an increased risk for subsequent infections, exposure to infection during the neonatal period is not associated with an increased risk of subsequent infection and may be paradoxically associated with reductions in late-onset sepsis (LOS) in the most premature infants. Perinatal inflammation is also associated with a decreased incidence of asthma and atopy later in life. Conversely, septic neonates are at increased risk of impaired long-term neurodevelopment. While the positive effects of antibiotics in the setting of infection are irrefutable, prolonged administration of broad-spectrum, empiric antibiotics in neonates without documented infection is associated with increased risk of LOS, necrotizing enterocolitis, or death. Vaccines provide a unique opportunity to prevent infection-associated disease; unfortunately, vaccinations have been largely unsuccessful when administered in the first month of life with the exception of vaccines against hepatitis B and tuberculosis. Future vaccines will require the use of novel adjuvants to overcome this challenge. This review describes the influence of infections, antibiotics, and vaccines during the first days of life, as well as the influence on future health and disease. We will also discuss potential immunomodulating therapies, which may serve to train the preterm immune system and reduce subsequent infectious burden without subjecting neonates to the risks accompanied by virulent pathogens.

## Introduction

Neonates, especially those born preterm (<37 weeks gestation), are prone to infections and sepsis given their diminished adaptive and innate immunity, decreased pro-inflammatory response, and attenuated antigen presentation and signaling (1). This unique immunological profile is possibly a result of the intrauterine fetal environment in which there is a need for immune tolerance to maternal antigens (2); however, this lack of a substantial immune response places the neonate at significant risk to microbes in the extrauterine environment. In the United States, early-onset sepsis (EOS; defined as sepsis occurring in the first 72 h of life) occurs at approximately 0.76–1 case per 1,000 live births with an increased incidence among very low birth weight (VLWB; <1,500 g) and preterm neonates (3–6). Due to concerns for infectious complications among preterm neonates, empiric antibiotics are almost universally administered shortly after birth (7). Meanwhile, healthy term neonates are administered hepatitis B vaccination in the first month of life, generally during the birth hospitalization or first clinic visit. How the presence of infections as well as the use of antibiotics and vaccines in the early neonatal period influences future health and disease remains an extremely complex and expanding topic. Herein, we review the impact of early life immune system exposures and discuss the use of immunomodulatory therapies to positively augment host protective immunity.

## Early-Life Exposures

### Infections

Early-onset sepsis is generally acquired *via* maternal ascending vaginal infection (8) (Table 1). Untreated genital tract colonization with group B streptococcus (GBS), prolonged rupture of membranes, and chorioamnionitis are known risk factors for the development of EOS (9–12). Importantly, two large, retrospective studies of very low birth weight (VLBW) neonates failed to identify an association between the development of EOS and the risk for subsequent or late-onset sepsis (LOS; defined as sepsis occurring after 72 h of life) (13, 14). Interestingly, neonates who were born at <25 weeks gestation and survived EOS showed a significant reduction in risk of LOS or death by 120 days (14). Extremely preterm neonates that are able to survive EOS may simply have a more robust immune response and thus, have a bias to equally fair well with subsequent infections. An alternative explanation offered by the authors is that the early immune stimulus may transform the preterm neonatal immune system from a relative state of tolerance to a level of competence that is better suited to defend against pathogens in the extrauterine environment (14).

**Table 1:** Early-onset sepsis (EOS) and late-onset sepsis (LOS) characteristics.

	EOS	LOS
Age	<72 h	>72 h
Source	Maternal genital tract	Nosocomial
Pathogen	GBS, *Escherichia coli*	Coagulase-negative staphylococcus
Risk factors	Maternal infections, prolonged ROM, chorioamnionitis	Prolonged mechanical ventilation and intravascular access
Incidence	1.7% among VLBW neonates	21% among VLBW neonates

*GBS, group B streptococcus; ROM, rupture of membranes; VLBW, very low birth weight*.

*Incidence for EOS and LOS adapted from Ref. (3, 15), respectively*.

**Figure 1:**
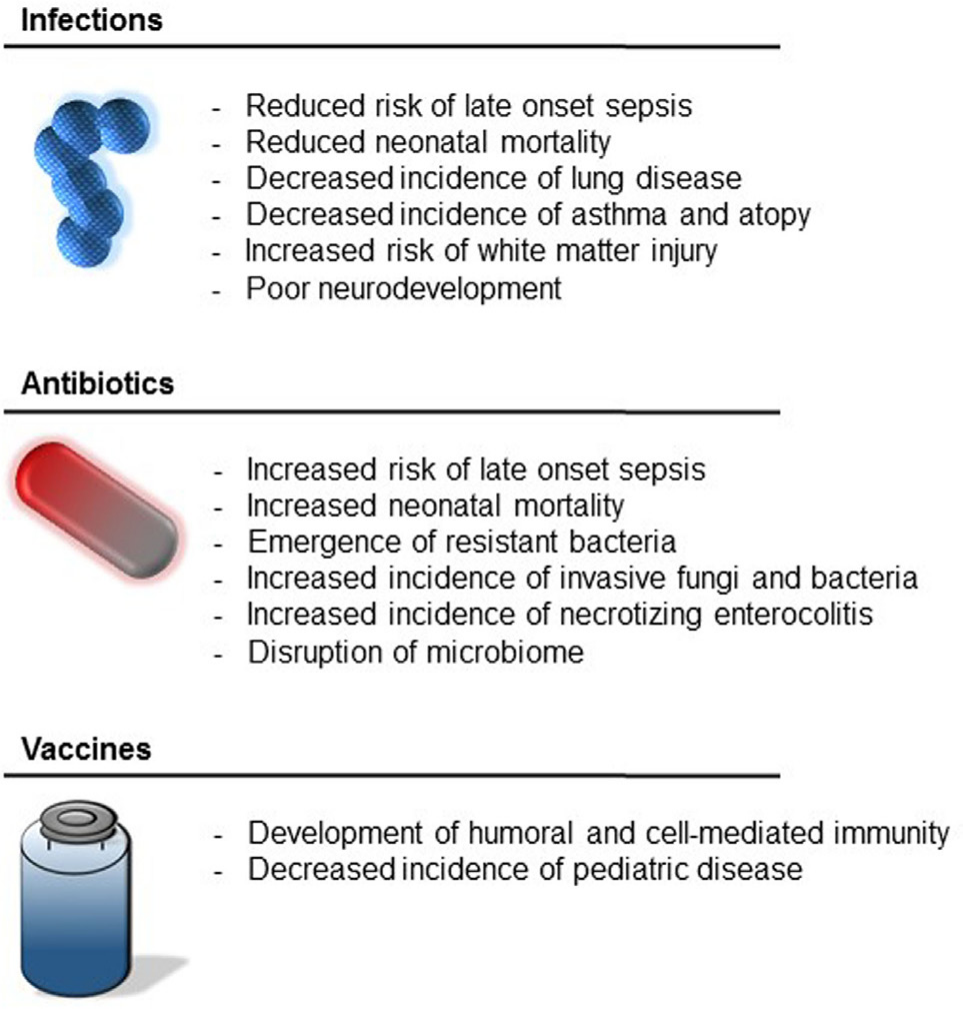
Impact of early-life exposures.

Chorioamnionitis likewise has been shown to be associated with reductions in respiratory distress syndrome, chronic lung disease, and mortality in preterm neonates (16–19) (Figure 1). The mechanism by which chorioamnionitis decreases the incidence of pulmonary disease in preterm neonates is likely *via* increased levels of interleukin (IL)-1 and IL-6, which stimulate pulmonary surfactant production and promote fetal lung maturation (20–23). Meanwhile, the observed difference in mortality among neonates born to mothers with chorioamnionitis may be explained in part by the fact that these neonates develop significantly fewer cases of LOS, which has been associated with prolonged hospital stays and death (15, 24).

The presence of early exposure to inflammation, bacteria, and infections may have lasting beneficial effects. Sepsis among preterm neonates appears protective to the development of childhood asthma (25). Birth cohort studies investigating the impact of endotoxin [bacterial lipopolysaccharide (LPS)] exposure during infancy on the risk of later wheezing, atopy, and asthma have had largely mixed results (26–32). More recently, Lynch et al. demonstrated that children who had reduced bacterial exposure in the first year of life were more likely to develop atopy at age 3 years (33). In the September 2015 issue of *Science*, Schuijs et al. published their results utilizing a murine model of asthma to investigate the impact of chronic exposure to low-dose endotoxin and concluded that endotoxin protects mice from asthma development by increasing the synthesis of the enzyme A20 (a nuclear factor-κB attenuator) in airway epithelial cells (34).

Early exposure to inflammation and infection is not without harmful and devastating effects. VLBW neonates with EOS are about threefold more likely to die than those without EOS with an overall mortality of 35–37% by 120 days (3, 35). Moreover, a meta-analysis of 17 studies demonstrated that sepsis was associated with poor long-term neurodevelopment among VLBW neonates including cerebral palsy (36). Likewise, chorioamnionitis appears to be associated with cystic periventricular leukomalacia in preterm neonates, encephalopathy in term neonates, and cerebral palsy in both preterm and term neonates (37–41). Injury to the preterm brain is believed to result from a multi-hit mechanism in which the neonate is first exposed to inflammation and cytokine release *in utero*, leading to increased susceptibility to subsequent perinatal and postnatal insults (42). This model is supported by the work of Korzeniewski et al. who demonstrated the cumulative contributions of chronic placental inflammation, acute fetal inflammation, and postnatal inflammatory events on neonatal white matter injury (43). Microglial activation has a central role in this process *via* excitotoxic, inflammatory, and free radical injury to the developing central nervous system (44). The aforementioned hypothesis has been further validated in rat models of the generation of neuroinflammation in which rat pups subjected to both endotoxin and hypoxic ischemia demonstrated white water injury but sham and endotoxin alone groups did not (45). The group that received both endotoxin and hypoxic ischemia notably had an increase in activated microglia and tumor necrosis factor (TNF)-alpha expression compared to the other groups. The investigation of potential therapies to treat theses prenatal insults by targeting activated microglia and astrocytes is ongoing and includes the administration of dendrimer-based *N*-acetyl-l-cysteine treatment in the postnatal period, which has been shown to suppress neuroinflammation and improve motor function in newborn rabbits with cerebral palsy (46).

### Antibiotics

The first exposure to antibiotics often occurs prior to birth in the form of intrapartum antibiotic prophylaxis against GBS, treatment of suspected chorioamnionitis, or antibiotic prophylaxis for women undergoing elective or emergency Cesarean sections. The use of intrapartum antibiotics is steadily increasing; for example, Van Dyke et al. demonstrated that the percentage of pregnant women receiving intrapartum increased from 26.8% in 1998–1999 to 31.7% in 2003–2004 (47). In an era of widespread prophylactic treatment of GBS for colonized pregnant women, the incidence of invasive early-onset GBS disease has decreased by more than 80%; however, the incidence of late-onset invasive GBS disease has remained unchanged (48). The use of maternal antibiotic prophylaxis is not without risks including the emergence of antimicrobial resistance invasive GBS and other neonatal pathogens. Between 1996 and 2003, clindamycin and erythromycin resistance has significantly increased in invasive GBS isolates (49). The incidence of early-onset *Escherichia coli* sepsis has also significantly increased in VLBW neonates with 64–85% of recent cases having resistance to ampicillin (35, 50). Not unexpectedly, neonates with ampicillin-resistant *E. coli* infections were more likely to be born from mothers who received intrapartum ampicillin (35, 50–52). Moreover, a multicenter case–control study during 1995–1996 demonstrated that cases of resistance *E. coli* infection were more often preterm (91 vs 20%, *p* < 0.001) and had significantly greater morality (40.9 vs 0%, *p* = 0.017), compared to cases of susceptible *E. coli* infections (51).

Among underweight and preterm neonates, the use of empiric antibiotics has essentially become standard of practice with antibiotics being the most prescribed medications in the neonatal intensive care unit (53). This phenomenon is largely due to the difficulty of accurately diagnosing neonatal sepsis in symptomatic neonates with developmental immaturity. A retrospective cohort analysis of 5,693 extremely low birth weight (ELBW; <1,000 g) neonates demonstrated that 98% of neonates received antibiotic treatment in the first three postnatal days, while <2% of neonates had positive blood cultures and clinical symptoms of EOS (7). The majority of neonates in the cohort received >5 days of empiric antibiotics despite having negative cultures; each additional day of empiric treatment was associated with a 4% increase in the odds of necrotizing enterocolitis (NEC) and a 16% increase in the odds of death (7). Similarly, Kuppala et al. demonstrated prolonged administration of empirical antibiotics was associated with increased LOS and the composite outcome of LOS, NEC, or death (54). These short-term deleterious outcomes, as well as an increased incidence of invasive candidiasis, may be the result of intestinal microbiome modification, including decreased microbial diversity, which is associated with broad-spectrum antibiotic use in ELBW and preterm neonates (55–57). The disruption of the microbiome may lead to long-term health consequences including decreased absorption of nutrients and vitamin production, as well as increased risk of infections, asthma, diabetes, and obesity. Further discussion on the effect of antibiotics on the microbiome and the role of dysbiosis in pediatric disease is beyond the scope of this mini review, but the interested reader is directed to a number of outstanding recent reviews (58–60).

### Vaccines

Vaccines provide a unique opportunity to prevent infection-associated disease. Hepatitis B vaccine is the only vaccine currently recommended in the first month of life by the United States Department of Health and Human Services, Centers for Disease Control and Prevention and is often administered during the birth hospitalization for healthy, term neonates (61, 62). Essentiality, all infants administrated hepatitis B vaccine respond with hepatitis B surface antigen-specific humoral and cell-mediated immunity following completion of the primary vaccine series (63). Although antibody titers decrease over time, immunological memory persists with vaccinated responders mounting a rapid anti-hepatitis B surface antibody response to a vaccine challenge (63). This immunological memory has had a dramatic impact on reducing hepatitis B infection and disease worldwide. After the implementation of universal hepatitis B vaccination program in Taiwan, the seroprevalence rate of hepatitis B surface antigen in children decreased from 10 to 0.7% (64). Likewise, universal vaccination significantly reduced the incidence of pediatric fulminant hepatitis and hepatocellular carcinoma (64). In addition to its clear beneficial effects, early vaccination for hepatitis B remains remarkably safe. Over one billion doses of hepatitis B vaccine have been administered worldwide with few true adverse reactions, and no evidence of an association with sudden infant death syndrome, multiple sclerosis, or chronic fatigue syndrome (65).

Contrasting with the success of the hepatitis B vaccine, the use of other vaccines early in life has been more challenging and frequently less successful. The administration of vaccines against influenza, measles, and mumps during infancy has been unsuccessful given the poor generation of host antibodies (66, 67). Likewise, infants demonstrate decreased cell proliferation and IFN-γ production in response to the polio vaccine, compared to adults (68). This relative resistance to the development of life-long adaptive immunity early in life has impeded the use of many current vaccines in neonates. Generally, this has been attributed to the absence of a strong type 1 T helper cellular response to the antigen. The use of immune adjuvants appears to be one of the best methods to elicit a stronger immune response and overcome this limitation. Currently, aluminum salts, oil-in-water emulsions (MF59, AS03, AF03), virosomes, and AS04 [monophosphoryl lipid A (MPLA) preparation with aluminum salt] are being used as adjuvants in vaccines approved for use in the United States and/or Europe (69).

## Implications for Future Therapies

The first step in being able to combat invading pathogens relies on their proper recognition by host cellular populations. This occurs *via* complement in blood and pattern recognition receptors including toll-like receptors (TLRs), C-type lectin receptors, nucleotide-binding oligomerization domain (NOD)-like receptors, beta integrins, and others on cells responsible for immune surveillance (70). Specifically, TLRs are located on and within numerous cell populations, including immune, epithelial, and endothelial cell populations. TLRs continuously survey the environment to recognize microbial components and intracellular signals of infection and/or cellular damage. Activation leads to downstream signaling, transcriptional changes, and the eventual secretion of inflammatory cytokines, type I IFN, chemokines, and antimicrobial peptides, which together function to target, localize, and kill the invading pathogen (70). In neonatal murine models, CpG oligodeoxynucleotides (TLR 9 agonist) have shown promise in improving survival to *Listeria monocytogenes, Cryptosporidium parvum*, and neurotropic Tacaribe arenavirus infections (71–73). In addition, LPS (TLR 4 agonist) and resiquimod (TLR 7/8 agonist) were shown to augment innate immunity, reduce bacteremia, and improve survival to polymicrobial sepsis (74); nevertheless, LPS is highly toxic and thus not suitable for clinical use. In *ex vivo* human newborn cord blood studies, novel agonists VTX-294 (TLR 8 agonist) and Hybrid-2 (TLR 7/8 agonist) demonstrated a greater cytokine-inducing potency compared to resiquimod (75, 76). Moreover, VTX-294 acted in synergy with MPLA (TLR 4 agonist) to induce an even greater production of TNF and IL-1β (75). Finally, Dowling et al. recently demonstrated that the TLR 7/8 agonist 3M-052 synergistically enhances type 1 immunity from newborn leukocytes when combined with pneumococcal conjugate vaccine (PCV13) *in vitro* and accelerates neonatal serotype-specific antibody response and pneumococcal opsonophagocytic killing (77).

In addition to increasing the immune responsiveness to the targeted pathogen, the use of TLR agonists in vaccines may provide additional non-specific immune benefits. As a particular example, the bacillus Calmette–Guerin (BCG) vaccine against tuberculosis is the most commonly administered vaccine worldwide and possesses inherent TLR 2/4/8 activity (78). In under-resourced areas of the world, BCG vaccinations are frequently given to neonates on the day of birth due to the absence of consistent postnatal care. Neonatal BCG vaccination has been shown to induce an adult-like immune response characterized by a predominant production of IFN-γ by CD4^+^ T lymphocytes (79). Administration of BCG vaccine at birth in Guinea-Bissau led to a 41% reduction in all-cause mortality at 12 months among VLBW neonates (80). This reduction was attributed not to reduced tuberculosis but to fewer cases of neonatal sepsis and respiratory infections. It is likely that the success of this vaccine in early life is due to the induction of a strong immune response by the engagement of multiple TLRs simultaneously by products of the *Bacillus* (81). These findings require further investigation and may lead to the development of novel immune agonists that can augment the host immune response early in life with an associated reduction in the infectious burden in neonates. The human adult literature on the use of TLR agonists as modulators of the innate immune response and as therapeutic strategies for the management of sepsis is vast and beyond the scope of this mini review, but there are several recent outstanding reviews (82–84).

The basal expression of TLRs, accessory proteins, and adaptor proteins on neonatal mononuclear cells is similar to adults; nevertheless, the early gene activation secondary to ligation of these receptors appears to be reduced in neonates due to impaired MyD88 and p38 signaling (85, 86). Understanding TLR biology is important for developing new compounds and ligands, which can activate these receptors and their signaling pathways. Alternative approaches using activators of the inflammasome in combination with TLR agonists may be considered. Future TLR ligands must be able to induce a sufficient immune response while remaining safe in newborns. This balance has made the development of innate immune agonists a difficult task. Therapeutic use of immunotherapies with agonists, which result in the development of antimicrobial resistance, holds great promise to be used prophylactically in the most susceptible population (i.e., VLBW and preterm neonates), in combination with live attenuated organisms to foster development of long-lasting antigen-specific immunity. As mentioned previously, engagement of multiple TLRs at the same time brings greater proliferation and higher cytokine production; however, the clinical applications of TLRs agonists have been limited to local delivery to minimize immune response-related toxicity (87). New vaccine strategies taking advantage of the inclusion of TLR and NOD agonists are currently being investigated to activate dendritic cells, enhance antigen presentation, and improve the host protective immune response (88–93). These novel vaccines require further investigations particularly in the neonatal population to prevent and treat infectious diseases among our most vulnerable patients.

## Conclusion

The impact of infections, antibiotics, and vaccines during the early neonatal period, and their influence on future health and disease remains an important and evolving area of research. A better understanding of the immediate and long-term effects of these exposures may lead to novel therapeutics with the ability to drastically reduce infectious complications and mortality in the neonatal period as well as promote longstanding health.

## Author Contributions

SR, JR, JW, LM, and SL drafted and revised the manuscript.

## Conflict of Interest Statement

The authors declare that the research was conducted in the absence of any commercial or financial relationships that could be construed as a potential conflict of interest.
